# Simultaneous Detection of Carnosine and Anserine by UHPLC-MS/MS and Its Application on Biomarker Analysis for Differentiation of Meat and Bone Meal

**DOI:** 10.3390/molecules24020217

**Published:** 2019-01-09

**Authors:** Yahong Han, Bing Gao, Shengnan Zhao, Mengyan Wang, Lin Jian, Lujia Han, Xian Liu

**Affiliations:** College of Engineering, China Agricultural University, Beijing 100083, China; hyhmelody@cau.edu.cn (Y.H.); gaobing@cau.edu.cn (B.G.); zhaoshengnan@cau.edu.cn (S.Z.); wangmy@cau.edu.cn (M.W.); jianlin@cau.edu.cn (L.J.); hanlj@cau.edu.cn (L.H.)

**Keywords:** carnosine, anserine, UHPLC-MS/MS, meat and bone meal, biomarker

## Abstract

A novel ultra-high performance liquid chromatography (UHPLC) procedure, coupled with tandem mass spectrometry (MS/MS), was established for the analysis of anserine (ANS) and carnosine (CAR) in meat and bone meal (MBM) (bovine, ovine, porcine, and poultry origins). The pretreatment strategies were optimized for four types of MBM samples prior to UHPLC-MS/MS analysis. This method allowed determining CAR and ANS in short analysis time (18 min per sample). The limits of detection (LODs) and limits of quantification (LOQs) of two analytes in four types of MBM samples were in the ranges of 0.41–3.07 ng/g and 0.83–5.71 ng/g, respectively. The recovery rates spiked with low, intermediate, and high levels of two analytes in four types of MBM samples were 48.53–98.93%, 60.12–98.94%, and 67.90–98.92%, respectively. Acceptable inter-day reproducibility (RSD < 12.63%) supported the application of this proposed method for determining CAR and ANS in MBM samples. Overall, this rapid, effective, and robust method was successfully applied for quantitative detection of CAR and ANS in MBM samples. Furthermore, The CAR/ANS ratio was found to be in the decreasing order: porcine > bovine > ovine > poultry MBM. This proposed methodology was novelly applied to identify the biomarker (CAR/ANS ratio) for species-specific identification of MBM.

## 1. Introduction

Meat and bone meal (MBM) with the high protein amount, has been widely used in aquaculture. However, due to the outbreak of mad cow disease, the use of MBM was restricted by regulation globally [1,2,3]. In order to achieve the sustainable development of the feed industry, the development trend of animal-derived feed regulations could be to identify MBM from different sources [4,5]. Currently, polymerase chain reaction (PCR) and light microscopy are the only two methods authorized by the European Union [1]. The PCR technique, as the only official method for identifying the species, has its limitations, such as exogenous contamination and DNA degradation [6]. Consequently, it is indispensable to establish an alternative methodology for differentiating MBM from various species. 

The main component of MBM is protein (typically 45%–63%) [7,8]. It has been proven that the amino acid composition of various protein sources was different, such as the difference in amino acid composition between MBM and fish meal [9]. Consequently, an insight into the species-specific identification of MBM samples, based on the difference in amino acid composition or the proportion of specific amino acids, was proposed. 

The amounts of anserine (*N*-β-alanyl-3-methyl-l-histidine, ANS), and carnosine (β-alanyl-l-histidine, CAR) [10] vary with species. CAR and ANS have been proven to be absent in plant samples, such as soybean and malt seed, while they were found in animal tissues [11]. It was further revealed that the CAR/ANS ratio was less than 0.3 in fresh poultry meat, while the CAR/ANS ratio was higher than 0.3 for fresh mammal meat, such as pork, beef, and lamb [12]. The ratio was found to be 49 in fresh pork meat, while the ratio was 8 in fresh beef meat [13]. Therefore, a hypothesis has been proposed that the CAR/ANS ratio may be considered as a biomarker for discriminating MBM from various species. However, most of the experimental samples in the aforementioned literatures were concentrated on fresh meat tissue samples. The objective of MBM in the present study was quite different from fresh meat tissues, which is prepared by high temperature treatment (133 °C, 20 min, 300 kPa) of fresh meat and bone meal. It has been demonstrated that high-temperature treatment could significantly reduce the amount of CAR and ANS [14,15]. The amounts of CAR and ANS in beef were found to be significantly reduced to approximately 50% and 70% by high temperature treatment (100 °C, 10 min), respectively [14]. In addition, another difference is the composition of MBM, which is made up of bone (approximately 70%–90%) and meat (approximately 10%–30%). CAR and ANS were found to be especially rich in meat tissues [12,16,17]. These two factors together may have effects on CAR/ANS ratio in MBM samples. Therefore, it put forward higher requirements (more sensitivity and lower detection limits) to verify this hypothesis.

Currently, the analytical techniques for CAR and ANS in fresh meat tissues are high-performance liquid chromatography (HPLC) [12,13,18,19] with the detection system of ultraviolet [11,17,19], fluorescence [12], photodiode array [15,20], pre-column/post-column derivatization [21,22], and mass spectrometry (MS) [13,23,24]. However, no studies have been reported for simultaneously determining CAR and ANS in MBM. As mentioned above, MBM samples and fresh meat tissues have great difference in the content of CAR and ANS. Traditional HPLC method could not meet the analysis requirement of CAR and ANS in MBM due to its high limit of detection (LOD). It was confirmed by our preliminary experiment. Therefore, a novel ultra-high performance liquid chromatography tandem MS (UHPLC-MS/MS) method with low LOD and high resolution and sensitivity was developed to simultaneously determine the CAR and ANS in four types of MBM samples (bovine, ovine porcine, and poultry origins). Pretreatment strategies were optimized for four types of MBM to eliminate impurities and improve their recovery rates. This developed methodology was novelly applied to identify the biomarker (CAR/ANS ratio) for species-specific identification of MBM.

## 2. Results and Discussion 

### 2.1. UHPLC-MS/MS 

Initially, HPLC method with hydrophilic interaction chromatography (HILIC) column was applied for quantitative analysis of CAR and ANS in MBM samples, in accordance with the procedure of Mora et al. [18]. This method was widely used for the analysis of CAR and ANS in meat tissue [15,25]. However, the result was not satisfactory. On the one hand, CAR and ANS can’t be separated totally. It may be due to that the polarities of two analytes are similar. On the other hand, it was found that the amounts of ANS in some samples were lower than the LOD of this method (5.64 μg/mL for CAR and 8.23 μg/mL for ANS) [18]. It was because the high temperature treatment in the process of MBM could significantly decrease the amounts of CAR and ANS [14]. Therefore, it is indispensable to establish a UHPLC-MS/MS technology for the analysis of CAR and ANS in MBM samples.

**Figure 1:**
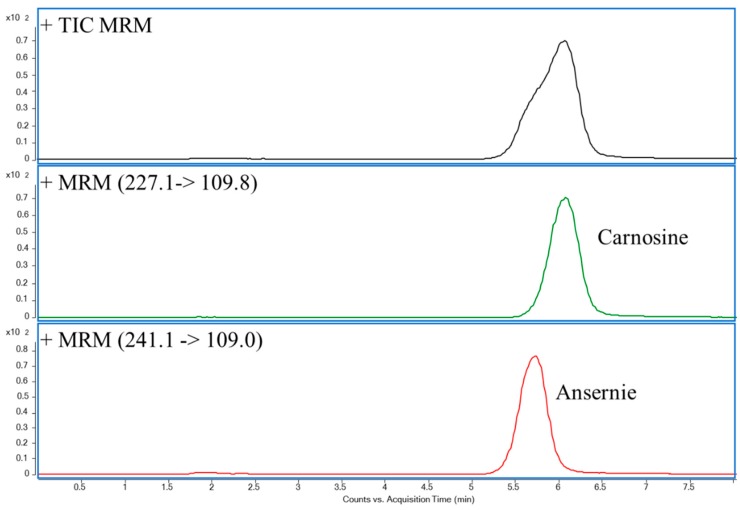
The MRM chromatograms of anserine and carnosine.

In order to optimize the separation efficiency and retention time of UHPLC columns, five candidate UHPLC columns, including Acquity UHPLC BEH AMIDE (150 × 2.1 mm, i.d., 1.7 μm), Acquity UHPLC CSH Fluoro Phenyl (150 × 2.1 mm, i.d., 1.7 μm), Acquity UHPLC HSS C18 (150 × 2.1 mm, i.d., 1.8 μm), Acquity UHPLC HSS T3 (150 × 2.1 mm, i.d., 1.8 μm), and Acquity UHPLC BEH Shield RP18 (150 × 2.1 mm, i.d., 1.7 μm), were employed. Supplementary Figure S1 summarizes UHPLC-MS/MS chromatograms of carnosine and anserine separated by five candidate UHPLC columns. Besides, the extracted ion chromatograms of CAR and ANS by ACQUITY UHPLC BEH AMIDE column were illustrated in Figure 1. It indicated that this column could be used to separate CAR and ANS. Therefore, in terms of the chromatographic retention of two analytes and separation effect between CAR and ANS, the Acquity UHPLC BEH AMIDE column was employed in this study.

The mobile phases were then optimized to obtain the best separation effect and improve ionization efficiency [26]. Five alternative ratios of 10 mmol/L of ammonium acetate to acetonitrile were investigated (50:50, 45:55, 40:60, 35:65 and 30:70). As presented in Supplementary Figure S2, the result showed that the peak shape performed by the ratio of 30:70 was the best. Meanwhile, 0.1% (*v*/*v*) methanoic acid was added to the 10 mmol/L of ammonium acetate solution to improve the peak shape and intensities [27]. Consequently, the mobile phase, combined 10 mmol/L of ammonium acetate (containing 0.1% (*v*/*v*) of methanoic acid) and acetonitrile (30:70, *v*/*v*) at a constant flow rate of 0.2 mL/min was selected as the proposed mobile phase. 

In the present study, the multiple reaction monitoring (MRM) mode was used for qualitative and quantitative ions. Initially, [M + H]^+^ and [M − H]^−^ modes were compared and the result showed that more relative abundance of CAR and ANS in [M + H]^+^ than in [M − H]^−^ mode. Therefore, [M + H]^+^ mode was employed. The optimized MS/MS parameters including MRM transitions, retention time, and optimized collision and fragmentor voltage are shown in Table 1. In addition, Supplementary Figure S3 shows the fragmentation patterns of CAR and ANS. Using the optimized UHPLC-MS/MS condition, the extracted ion chromatograms of CAR and ANS of an MBM sample is illustrated in Supplementary Figure S4. 

### 2.2. Optimization of Sample Pretreatment Procedure

**Figure 2:**
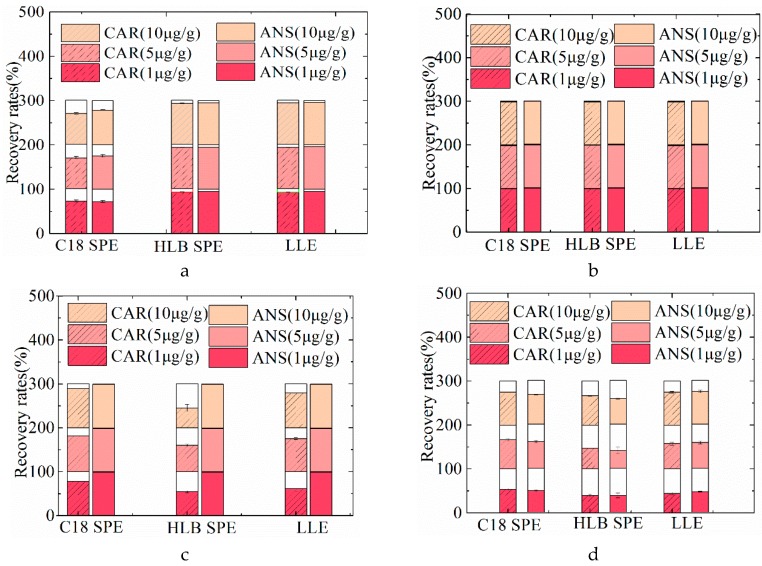
The recovery rates of CAR and ANS spiked with different concentrations (1, 5, and 10 μg/g) of standards using different extraction procedures (C18 solid-phase extraction (SPE), HLB SPE, and liquid-liquid extraction (LLE) procedures) in porcine (**a**), poultry (**b**), bovine (**c**), and ovine (**d**) MBM samples.

In order to purify samples, a sample pretreatment is of essential importance prior to the UHPLC-MS/MS analysis. Two solid-phase extraction (SPE) cartridge candidates, including Oasis^®^HLB cartridge [23] and Supelclean^TM^ LC-18 cartridge [28,29] were employed. Four types of MBM samples spiked with CAR and ANS standards (1, 5, and 10 μg/g for each compound) were treated with two SPE procedures to test the extraction recovery rates. In addition, a liquid-liquid extraction (LLE) procedure was conducted as a control group. The procedure was as follows: briefly, when the sample was homogenized with hydrochloric acid (Section 3.3), the homogenized solution and an equal volume of chloroform were added to the separation funnel, shaken and separated. It was repeated three times. The recovery rates of two analytes with three pretreatment procedures are illustrated in Figure 2 and Supplementary Table S1. 

For porcine MBM samples, CAR and ANS exerted higher extraction recovery rate (92.88–95.22%) when Oasis HLB was employed than recovery rate (69.73–78.68%) when C18 was used. For bovine MBM samples, the recoveries of two analytes, pretreated with C18 and Oasis HLB were 72.39–91.48% and 30.05–60.30%, respectively. For ovine MBM samples, the recoveries of two analytes, pretreated with C18 and Oasis HLB, were 48.53–75.05% and 37.76–66.67%, respectively. It was concluded that two analytes from bovine and ovine MBM samples showed lower recovery when Oasis HLB was employed than recovery when C18 was used. For poultry MBM sample, the recovery rate of two analytes pretreated with C18 SPE purification was 98.82–98.94%, and when pretreated with Oasis HLB was 98.86–98.93%. It was found that the recovery rates of C18 and Oasis HLB SPE in poultry MBM samples are very similar. Considering the cost of SPE procedures, C18 SPE was finally selected to purify poultry MBM samples. In addition, an LLE procedure was employed for all of four species as a comparison of SPE procedures. The recovery of two analytes from porcine, poultry, bovine, and ovine MBM samples with liquid-liquid extraction procedure were 92.51–96.30%, 98.74%–98.97%, 40.63–82.37%, and 44.37–74.70%, respectively. Furthermore, it was found that the recovery rate with optimized SPE procedures were a little higher or similar to the recoveries with LLE procedure. It indicated that the extraction recovery rate with the SPE procedure was reliable. However, the LLE procedure has several limits, such as being time consuming and having a high solvent requirement [27]. Generally, C18 SPE was used for purifying poultry, bovine, and ovine MBM samples, while HLB SPE was used for porcine MBM samples.

### 2.3. Method Performance

Initially, animal matrix standard addition curves (porcine, poultry, bovine, and ovine MBM) were built to eliminate the matrix effect. The results showed that the bovine matrix standard addition curve for CAR and ANS were *y* = 3.88 *x* + 12678.24 and *y* = 1.23 *x* + 734.82, respectively, while the ovine matrix standard addition curve for CAR and ANS were *y* =18.80 *x* + 12243.38 and *y* = 4.25 *x* + 4295.11, respectively (Table 2). However, the poultry matrix standard addition curves for two analytes could not form straight lines. What’s more, the porcine matrix standard addition curve for CAR was not a straight line either. It may be due to higher amounts of CAR and ANS in porcine and poultry MBM samples, because porcine and poultry MBM samples contain more meat components. Therefore, a simulated matrix without CAR and ANS was used to replace MBM to establish a matrix standard addition curve. In order to obtain precise result, six calibration curves, including a simulated matrix standard additional curve, a solution standard curve, and four animal matrix standard addition curves were compared. As presented in Table 2, the slope of a simulated matrix standard additional curve for CAR and ANS were 10.84 and 6.51, respectively, while the slope of solution standard curve for CAR and ANS were 702.74 and 215.28, respectively. It was found that the difference between the slope of the animal matrix (bovine and ovine) and simulated matrix standard addition curves was smaller than that in the animal matrix (bovine and ovine) standard addition curve and the solution standard curve. Furthermore, intercepts of all calibration curves are positive except for the solution standard curve. Therefore, a simulated matrix standard addition curve was selected to determine the amounts of CAR and ANS in MBM samples. The linearity range of this calibration curve was from 10 to 2000 ng/mL. 

In addition, other instrumental quality parameters, including LOD, limit of quantification (LOQ), repeatability, and recovery rates are summarized in Table 3. LODs and LOQs were calculated in accordance with the signal-to-noise ratio criterion of three and ten, respectively. As a result, the LODs of two analytes in four MBM samples ranged from 0.41 to 3.07 ng/g, while the LOQs ranged from 0.83 to 5.71 ng/g. Compared with the method for determining CAR and ANS in meat tissues (2.63 ppm for CAR; 0.58 ppm for ANS) [12], this developed method has lower detection limits.

Furthermore, the repeatability was evaluated by the intra- and inter-day precision, in accordance with the ISO 5725 criteria [30]. The precision test was measured by the relative standard deviation (% RSD), in terms of four types of MBM samples spiked with CAR and ANS standard mixture solution at different levels (1, 5, and 10 μg/g). The intra- and inter-day precision tests were performed with 6 replicates within a day and 18 replicates over three days. RSD of intra- and inter-day precision tests were 1.34–7.16% and 2.40–6.54% in porcine MBM samples, 1.31–3.29% and 2.72–3.91% in poultry MBM samples. This result was smaller than the RSD ranges in the literature [23]. In addition, the RSD of intra- and inter-day precision tests were 2.21–10.17% and 5.69–8.74% in bovine MBM samples, 1.25–8.39% and 8.31–12.63% in ovine MBM samples. This confirmed the stability of the proposed UHPLC-MS/MS method. 

In summary, the current UHPLC-MS/MS methodology was validated to be sensitive, effective, and reliable, and it can be applied for the simultaneous analysis of CAR and ANS in four types of MBM samples.

### 2.4. Quantification of CAR and ANS in Meat and Bone Meal

**Figure 3:**
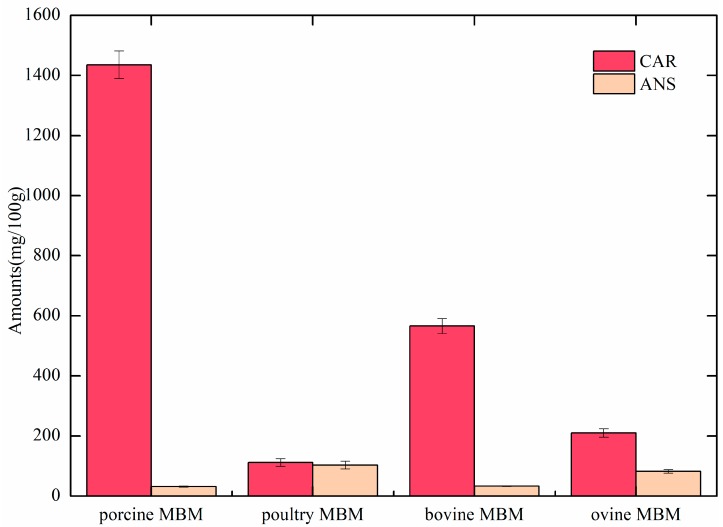
The amounts of carnosine (CAR) and anserine (ANS) in four types of MBM samples (porcine, poultry, bovine, and ovine origins).

The current UHPLC-MS/MS method was applied to quantify CAR and ANS in four types of MBM samples (bovine, ovine, porcine, and poultry origins). The result showed that this developed method could meet the requirements for determining CAR and ANS in MBM samples, in spite of the difference in amounts of two analytes, from 1.33 to 2862.35 mg/100g, which are depicted in Figure 3. 

**Figure 4:**
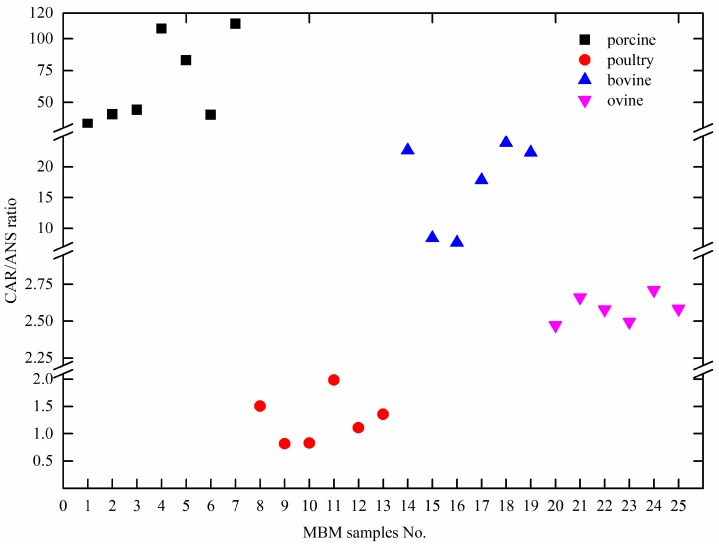
The carnosine amounts/anserine amounts (CAR/ANS) ratio in four types of MBM samples (porcine (No.1–No.7), poultry (No. 8–No. 13), bovine (No. 14–No. 19), and ovine origins (No. 20–No. 25)).

Compared with the study by Peiretti [13], the amounts of two analytes in MBM were lower than that in fresh meat. It may be due to high-temperature treatment and the different composition between MBM and fresh meat. Furthermore, it was found that CAR amounts were in the following order: porcine > bovine > ovine > poultry, while the ANS amounts were in the following order: porcine < bovine < ovine < poultry. This phenomenon was similar to the report by Aristoy [22], who suggested the same order in low-price muscle tissues. Results of the CAR and ANS ratio in four types of MBM samples are illustrated in Figure 4. The CAR/ANS ratio in porcine, poultry, bovine, and ovine MBM samples ranged from 33.17–111.63, 0.82–1.98, 7.69–23.90, and 2.47–2.71, respectively. The CAR/ANS ratios of four types of MBM were distributed in different ranges without overlapping. It could be concluded that this CAR/ANS ratio was in the decreasing order: porcine > bovine > ovine > poultry MBM. The same order was also revealed by Peiretti [13], who found that the CAR/ANS ratio was higher in pork than that in beef. Therefore, it was concluded that the CAR/ANS ratio could be considered as a biomarker for species-specific identification of MBM samples. 

## 3. Materials and Methods 

### 3.1. Chemicals

ANS and CAR standards were obtained from Sigma-Aldrich (St. Louis, MI, USA). Hydrochloric acid, chloroform, and ammonium acetate were analytical grade reagents provided by Sinopharm Chemical Reagent Beijing Co. Ltd. (Beijing, China). Acetonitrile and methanol were HPLC grade reagents purchased from Merck (Darmstadt, Germany). Ultrapure water was provided by a Milli-Q ultrapure system (Millipore, Bedford, MA, USA).

Stock solutions of CAR and ANS were prepared in acetonitrile/ultrapure water (70/30, *v*/*v*) at a concentration of 1 mg/mL and a standard stock mix of two standards was prepared weekly in acetonitrile/ultrapure water (70/30, *v*/*v*) at a concentration of 10 μg/mL.

### 3.2. Sample Preparation 

Bovine, ovine, porcine, and poultry neck bone and meat tissues were purchased from three local markets in Beijing. A total of 25 MBM samples, including bovine, ovine, porcine, and poultry origins, were prepared in the lab under the processing condition (133 °C, 20 min, 3 bar), in accordance with the European Commission [31]. All of the samples were ground with a sieve of 0.5 mm (ZM 200, Retsch, Germany) and then characterized by the real-time PCR method to guarantee species identity [32]. All of the MBM samples were then stored at −20°C until analysis. 

### 3.3. Sample Pretreatment

One gram of MBM sample was homogenized with 10 mL of 0.01 mol/L HCl in a centrifuge tube at 13,500 rpm for 1 min (T17, IKA, Staufen, Germany). The homogenate and 10 mL of chloroform were mixed and then centrifuged at 8000 rpm for 5 min at 4 °C. The supernatant was used for SPE purification procedure. 

For porcine MBM, the OASIS HLB Cartridge (60 mg; Waters Corp., Milford, MA, USA) was employed for supernatant purification. Prior to purification, the HLB cartridge was activated by 3 mL of methanol and 3 mL of deionized water in sequence. Two mL of the sample supernatant was loaded into the cartridge and collected. The collected solution was diluted 200-fold in the initial mobile phase and then filtered with the 0.22 μm filter submitted to UHPLC-MS/MS analysis. For poultry, bovine and ovine MBM, the Supelclean^TM^ LC-18 Cartridge (500 mg; Supelco, Bellefonte, PE, USA) was used, which was activated by 3 mL of methanol and 3 mL of deionized water in sequence. Then, 2 mL of the sample supernatants were loaded into the cartridge and collected. The collected solutions were then diluted with the initial mobile phase (poultry MBM 2000-fold; bovine and ovine MBM, 20-fold). The diluted solutions were finally filtered with a 0.22 μm filter and submitted to UHPLC-MS/MS analysis.

### 3.4. UHPLC-MS/MS Analysis

The UHPLC analysis of CAR and ANS in the MBM samples was performed using a 1200 infinity quaternary UHPLC system (Agilent Technologies, Santa Clara, CA, USA). The chromatographic separation was optimally performed on an Acquity UPLC BEH Amide column (2.1 × 150 mm i.d., 1.7 μm). The mobile phase was an ammonium buffer solution (10 mmol/L ammonium acetate and 0.1% (*v*/*v*) methanoic acid) and acetonitrile (30:70, *v*/*v*) at a constant flow rate of 0.2 mL/min. The chromatographic column was maintained at 40 °C with a total run time of 18 min per samples. The sample injection volume was 5 μL.

Tandem mass spectrometry was performed on a 6460 triple-quadrupole mass spectrometer with ESI source (Agilent Technologies, Santa Clara, CA, USA). CAR and ANS were detected with the positive ion [M + H]^+^ channels under the multiple reaction monitoring (MRM) mode. The ionization working instrument parameters were depicted as follows: capillary voltage, 3.5 kV; sheath gas temperature, 375 °C; sheath gas flow, 8 L/min; spray head voltage, 500 V; atomization device pressure, 45 psi; dryer temperature, 300 °C; dryer flow, 5 L/min. N_2_ and Ar were used as the nebulizer and collision gases, respectively. Table 1 summarizes MRM transitions for qualitative and quantitative ion, the retention time, optimized collision and fragmentor voltage of CAR and ANS.

### 3.5. Quantitative Determination of CAR and ANS 

In order to determine the amount of CAR and ANS in MBM samples, a six-point calibration curve was constructed using a simulated matrix (mainly soy protein, fat, ash, originally without CAR and ANS) spiked with the mix of standard solution at the following concentrations: 10, 100, 200, 500, 1000, 2000 ng/mL. Furthermore, the amounts of CAR and ANS were calculated as follows:

(1) The amount of CAR (mg100g)=CCAR×V×f10000×m

(2) The amount of ANS (mg100g)=CANS×V×f10000×m

Here, *C_CAR_* and *C_ANS_* were the concentrations of the CAR and ANS for UHPLC-MS/MS analysis, respectively. The V denotes the volume of HCl solution (10 mL), f denotes the dilution ratio of different MBM samples (porcine MBM, 200; poultry MBM, 2000; bovine and ovine MBM, 20), and m denotes the weight of the MBM sample.

### 3.6. Method Validation 

The methodological validation was determined in accordance with ISO standard procedure, including calibration curve, linearity, LOD, LOQ, within-laboratory repeatability (intra and inter-day), and recovery tests [30,33]. 

## 4. Conclusions

The proposed UHPLC-MS/MS method, based on the ACQUITY UHPLC BEH AMIDE column, could be applied for analysis of CAR and ANS in four types of MBM samples within a short analysis time (18 min). A novel and effective purification procedure (HLB SPE for porcine MBM, C18 SPE for others) was optimized. The recovery rates with different spiking levels (1, 5, and 10 μg/g) were 48.53%–98.93%, 60.12%–98.94%, and 67.90%–98.92%, respectively. Also, this method, with high sensitivity (LOD 0.41–3.07 ng/g, LOQ 0.83–5.71 ng/g) and reproducibility (RSD < 12.63%), meets the requirements for quantitative detection of CAR and ANS in MBM. Furthermore, this method can be successfully applied for species-specific identification of MBM samples.

## Figures and Tables

**Table 1:** Retention time (t_R_), MRM transitions, optimized fragmentor and collision voltages of carnosine and anserine.

Compound	t_R_ (min)	Fragmentor (V)	Quantitative Ion Channel		Qualitative Ion Channel	
			Transition [M + H]^+^	Collision (V)	Transition [M + H]^+^	Collision (V)
Carnosine	15.0	110	109.8	20	156.0	20
Anserine	13.5	110	109.0	20	170.1	20

**Table 2:** Calibration curves of quantitative determination of carnosine and anserine in meat and bone meal samples.

	Carnosine				Anserine			
	Calibration Curve	SD of Slope	SD of Intercept	R^2^	Calibration Curve	SD of Slope	SD of Intercept	R^2^
Simulated matrix standard additional curve	*y* = 10.84*x* + 2864.84	0.38	350.54	0.995	*y* = 6.51*x* + 236.73	0.06	55.40	0.999
Solution standard curve	*y* = 702.74*x* − 22578.20	11.96	2016.25	0.999	*y* = 215.28*x* − 1140.49	7.39	429.42	0.996
Bovine matrix standard addition curve	*y* = 3.88*x* + 12678.24	0.42	393.05	0.955	*y* = 1.23*x* + 734.82	0.09	83.09	0.979
Ovine matrix standard addition curve	*y* = 18.8*x* + 12243.38	0.874	801.98	0.991	*y* = 4.25*x* + 4295.11	0.16	150.86	0.994
Porcine matrix standard addition curve	--	--	--	--	*y* = 1.22*x* + 3774.81	0.17	87.68	0.944
Poultry matrix standard addition curve	--	--	--	--	--	--	--	--

SD refers standard error; R^2^ refers to determination coefficient; -- refers none.

**Table 3:** Instrumental quality parameters of the developed method to determine carnosine (CAR) and anserine (ANS) in four types of MBM samples.

MBM Samples	LOD & LOQ (ng/g)	Spiking (μg/g)	CAR			ANS		
			Intra-Day Precision (*n* = 6%)	Inter-Day Precision (*n* = 18%)	Recovery (*n* = 18%)	Intra-Day Precision (*n* = 6%)	Inter-Day Precision (*n* = 18%)	Recovery (*n* = 18%)
Porcine MBM	LOD:0.91	1	1.34	3.06	92.89 ± 0.09	5.42	6.26	94.68 ± 0.28
	LOQ:2.73	5	2.81	4.08	93.35 ± 0.19	1.59	2.40	95.15 ± 0.07
		10	4.39	3.66	92.88 ± 0.35	7.16	6.54	95.22 ± 0.36
Poultry MBM	LOD:0.41	1	1.79	2.72	98.82 ± 0.02	1.31	3.55	98.93 ± 0.01
	LOQ:0.83	5	2.56	2.82	98.86 ± 0.03	1.57	3.04	98.94 ± 0.02
		10	3.29	3.91	98.85 ± 0.04	1.49	3.31	98.92 ± 0.02
Bovine MBM	LOD:1.43	1	3.91	6.99	78.24 ± 0.48	5.42	5.69	72.39 ± 1.66
	LOQ:4.78	5	2.21	7.47	81.21 ± 0.42	10.17	5.88	84.54 ± 1.44
		10	5.72	8.44	89.35 ± 0.62	4.86	8.74	91.48 ± 0.42
Ovine MBM	LOD:3.07	1	4.72	10.5	53.65 ± 2.10	3.6	12.63	48.53 ± 1.91
	LOQ:5.71	5	8.39	8.31	66.41 ± 2.68	7.96	8.68	60.12 ± 3.03
		10	1.25	11.82	75.05 ± 0.32	3.14	9.16	67.90 ± 1.01

LOD refers to limit of detection; LOQ refers to limit of quantification.

## Supplementary Materials

The following are available online. Figure S1: UHPLC-MS/MS chromatograms of carnosine and anserine separated by different UHPLC column lengths, diameters and partial sized. The chromatographic separation was performed on (A) Acquity UHPLC BEH AMIDE column (150 × 2.1 mm, 1.7 μm); (B) Acquity UHPLC CSH Fluoro Phenyl column (150 × 2.1 mm, 1.7 μm); (C) Acquity UHPLC HSS C18 column (150 × 2.1 mm, 1.8 μm); (D) Acquity UHPLC HSS T3 column (150 × 2.1 mm, 1.8 μm); (E) Acquity UHPLC BEH Shield RP18 column (150 × 2.1 mm, 1.7 μm); Figure S2: The comparison among separation of carnosine and anserine with five alternative ratios of 10 mmol/L of ammonium acetate to acetonitrile were investigated (50:50 (A), 45:55 (B), 40:60 (C), 35:65 (D) and 30:70 (E)); Figure S3: The mass spectrum of carnosine (A) and anserine (B); Figure S4: The MRM chromatograms of anserine and carnosine of a MBM sample; Table S1: Reproducibility and spiking recovery tests of carnosine (CAR) and anserine (ANS) in four types of MBM samples (bovine, ovine, porcine, and poultry origins).
