# Supplementary material for: Simultaneous Detection of Carnosine and Anserine by UHPLC-MS/MS and Its Application on Biomarker Analysis for Differentiation of Meat and Bone Meal
Source: Molecules. 2019 Jan 9;24(2):217. doi: 10.3390/molecules24020217 (PMC6359308; doi:10.3390/molecules24020217)
Supplement: Supplementary file 1 [file molecules-24-00217-s001.pdf]

Supplementary Material

Simultaneous detection of carnosine and anserine by UHPLC-MS/MS and its application on biomarker analysis for differentiation of meat and bone meal

Yahong Han, Bing Gao, Shengnan Zhao, Mengyan Wang, Lin Jian, Lujia Han, Xian Liu*

This supplementary information contains Supplementary Figure S1-S4 and Supplementary Table S1

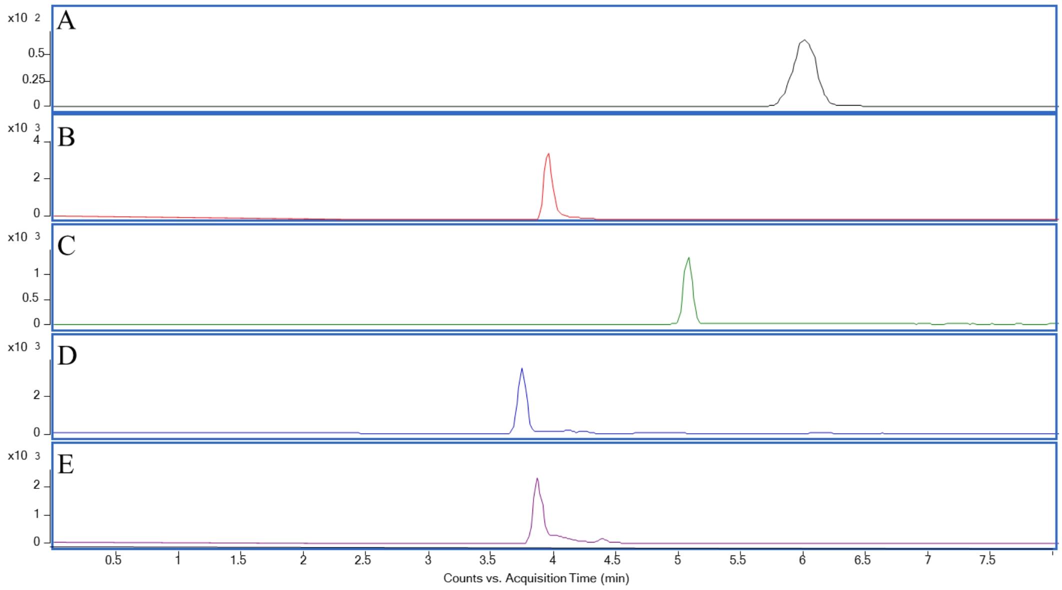

Supplementary Figure S1. UHPLC-MS/MS chromatograms of carnosine and anserine separated by different UHPLC column lengths, diameters and partial sized. The chromatographic separation was performed on (A) Acquity UHPLC BEH AMIDE column (150 × 2.1 mm, 1.7 µm); (B) Acquity UHPLC CSH Fluoro Phenyl column (150 × 2.1 mm, 1.7 µm); (C) Acquity UHPLC HSS C18 column (150 × 2.1 mm, 1.8 µm); (D) Acquity UHPLC HSS T3 column (150 × 2.1 mm, 1.8 µm); (E) Acquity UHPLC BEH Shield RP18 column (150 × 2.1 mm, 1.7 µm)

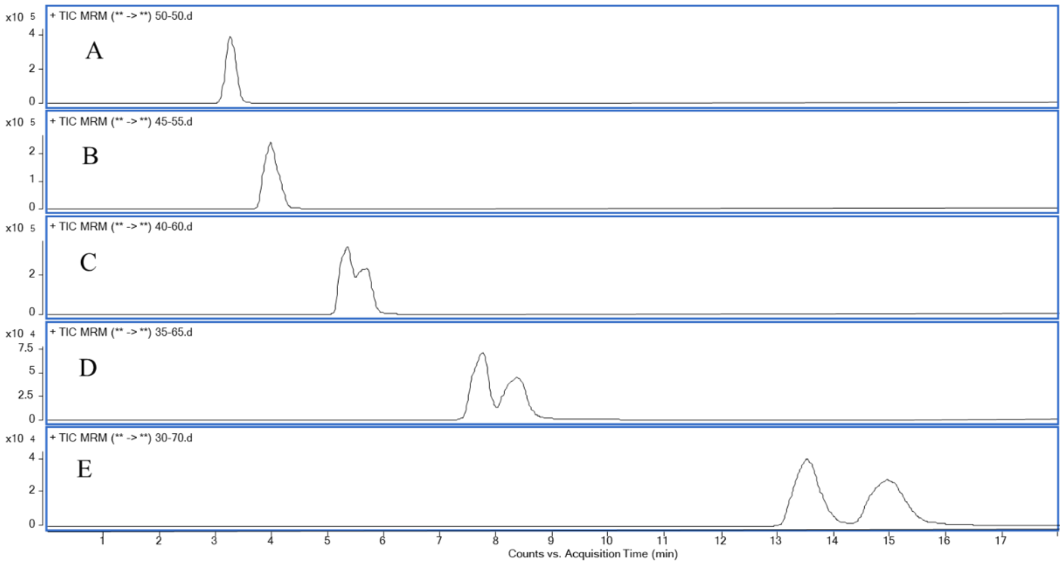

Supplementary Figure S2. The comparison among separation of carnosine and anserine with five alternative ratios of 10 mmol/L of ammonium acetate to acetonitrile were investigated (50：50 (A), 45：55 (B), 40:60 (C), 35:65 (D) and 30:70 (E))

(A)

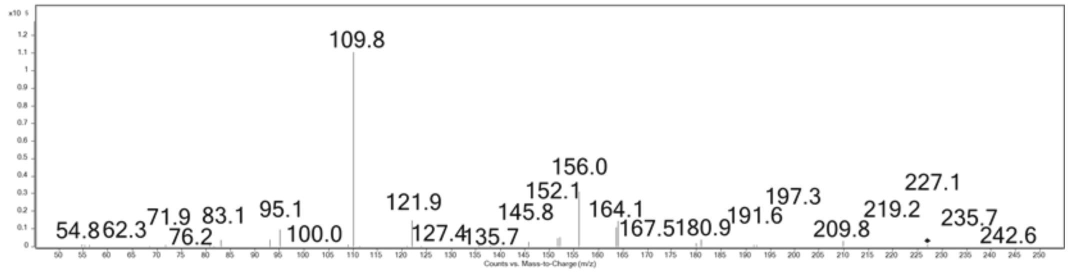

(B)

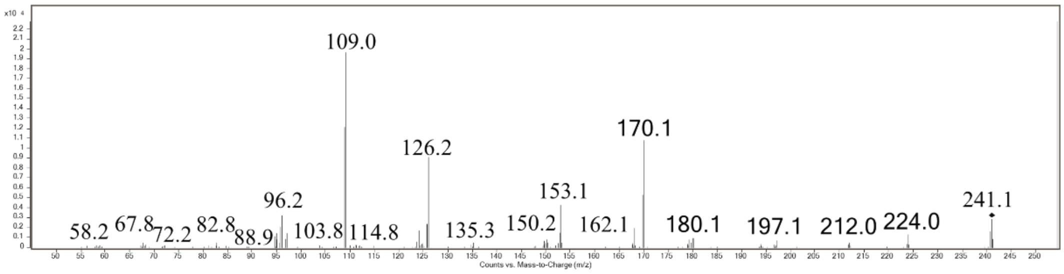

Supplementary Figure S3. The mass spectrum of carnosine (A) and anserine (B)

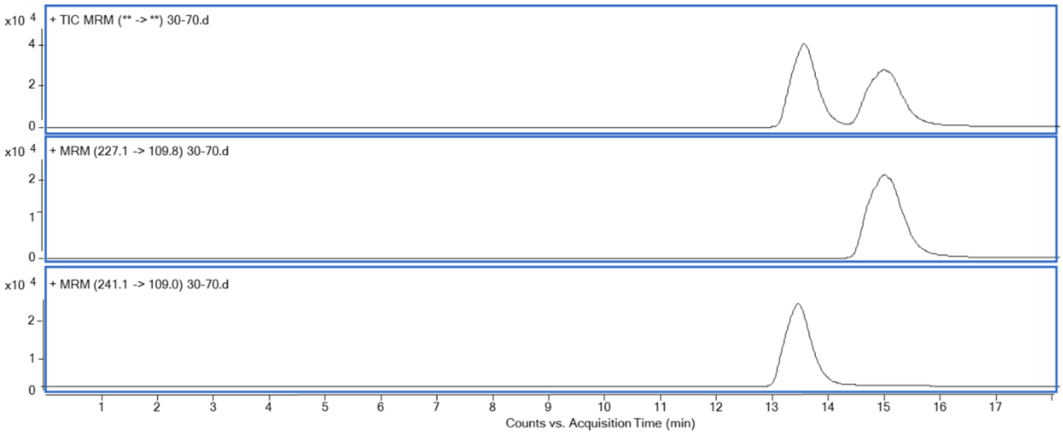

Supplementary Figure S4. The MRM chromatograms of anserine and carnosine of a MBM sample

Supplementary Table S1. Reproducibility and spiking recovery tests of carnosine (CAR) and anserine (ANS) in four types of MBM samples (porcine, poultry, bovine and ovine MBM samples) with different procedures (C18 solid phase extraction procedure (A), HLB solid phase extraction procedure (B), and liquid-liquid extraction procedure (C))

(A)

MBM samples	Spiking (μg/g)	CAR			ANS		
		Intra-day precision (n=6, %)	Inter-day precision (n=18, %)	Recovery (n=18, %)	Intra-day precision (n=6, %)	Inter-day precision (n=18, %)	Recovery (n=18, %)
Porcine MBM	1.00	9.57	4.51	72.39 ± 2.69	8.71	6.60	72.41 ± 2.47
	5.00	10.48	8.83	69.94 ± 3.05	11.76	7.28	75.29 ± 2.83
	10.00	6.79	4.55	69.73 ± 2.05	4.22	3.42	78.68 ± 0.91
Poultry MBM	1.00	1.79	2.72	98.82 ± 0.02	1.31	3.55	98.93 ± 0.01
	5.00	2.56	2.82	98.86 ± 0.03	1.57	3.04	98.94 ± 0.02
	10.00	3.29	3.91	98.85 ± 0.04	1.49	3.31	98.92 ± 0.02
Bovine MBM	1.00	3.91	6.99	78.24 ± 0.48	5.42	5.69	72.39 ± 1.66
	5.00	2.21	7.47	81.21 ± 0.42	10.17	5.88	84.54 ± 1.44
	10.00	5.72	8.44	89.35 ± 0.62	4.86	8.74	91.48 ± 0.42
Ovine MBM	1.00	4.72	10.50	53.65 ± 2.10	3.60	12.63	48.53 ± 1.91
	5.00	8.39	8.31	66.41 ± 2.68	7.96	8.68	60.12 ± 3.03
	10.00	1.25	11.82	75.05 ± 0.32	3.14	9.16	67.90 ± 1.01

(B)

MBM samples	Spiking (μg/g)	CAR			ANS		
		Intra-day precision (n=6, %)	Inter-day precision (n=18, %)	Recovery (n=18, %)	Intra-day precision (n=6, %)	Inter-day precision (n=18, %)	Recovery (n=18, %)
Porcine MBM	1.00	1.34	3.06	92.89 ± 0.09	5.42	6.26	94.68 ± 0.28
	5.00	2.81	4.08	93.35 ± 0.19	1.59	2.40	95.15 ± 0.07
	10.00	4.39	3.66	92.88 ± 0.35	7.16	6.54	95.22 ± 0.36
Poultry MBM	1.00	5.24	9.38	98.86 ± 0.05	1.08	1.27	98.91± 0.01
	5.00	3.60	3.89	98.87 ± 0.04	3.51	3.21	98.93 ± 0.04
	10.00	1.75	2.66	98.86 ± 0.02	0.69	2.08	98.90 ± 0.01
Bovine MBM	1.00	5.10	7.53	54.15 ± 2.58	6.78	10.15	30.05 ± 4.02
	5.00	6.67	6.43	60.30 ± 2.71	8.93	10.24	56.39 ± 3.71
	10.00	1.41	5.72	45.57 ± 7.80	1.34	7.98	52.79 ± 0.63
Ovine MBM	1.00	5.39	7.68	39.60 ± 3.24	9.26	9.84	37.76 ± 5.67
	5.00	1.21	4.62	47.36 ± 0.64	1.26	6.44	40.00 ± 7.50
	10.00	2.62	4.04	66.67 ± 0.90	3.96	4.90	58.67 ± 1.57

(C)

MBM samples	Spiking (μg/g)	CAR			ANS		
		Intra-day precision (n=6, %)	Inter-day precision (n=18, %)	Recovery (n=18, %)	Intra-day precision (n=6, %)	Inter-day precision (n=18, %)	Recovery (n=18, %)
Porcine MBM	1.00	2.26	10.72	92.51 ± 0.17	6.50	8.94	94.81 ± 0.03
	5.00	5.84	6.78	93.26 ± 0.39	11.49	10.52	95.89 ± 0.04
	10.00	2.29	7.27	93.88 ± 0.14	5.96	10.68	96.30 ± 0.22
Poultry MBM	1.00	14.26	9.85	98.74 ± 0.21	5.60	5.08	98.97 ± 0.06
	5.00	2.69	3.30	98.86 ± 0.03	3.01	2.70	98.89 ± 0.03
	10.00	2.33	3.13	98.81 ± 0.02	2.78	2.58	98.85 ± 0.03
Bovine MBM	1.00	16.27	10.67	61.22 ± 6.81	17.70	14.39	40.63 ± 11.69
	5.00	7.76	9.32	75.31 ± 1.96	10.11	11.32	72.04 ± 2.92
	10.00	2.74	6.80	79.73 ± 0.55	3.57	6.39	82.37 ± 0.64
Ovine MBM	1.00	5.14	5.74	44.37 ± 2.89	2.31	6.89	45.95 ± 1.25
	5.00	8.18	6.46	57.75 ± 3.29	7.93	9.61	58.16 ± 3.18
	10.00	8.41	7.90	74.70 ± 2.02	12.53	10.87	74.58 ± 2.96
